# Supplementary material for: Conditional chemoconnectomics (cCCTomics) as a strategy for efficient and conditional targeting of chemical transmission
Source: eLife. 2024 Apr 30;12:RP91927. doi: 10.7554/eLife.91927 (PMC11060718; doi:10.7554/eLife.91927)
Supplement: Figure 3—source data 3. [file elife-91927-fig3-data3.zip › Figure 3 source data3 part 1.zip!/Figure 3-Source data 3-part1/1081-1-CRISPResso_Report_8zosk6yj.zip!/CRISPResso_on_1081_1/9.Alleles_frequency_table_around_sgRNA_tttctggactacagtcccca.pdf]

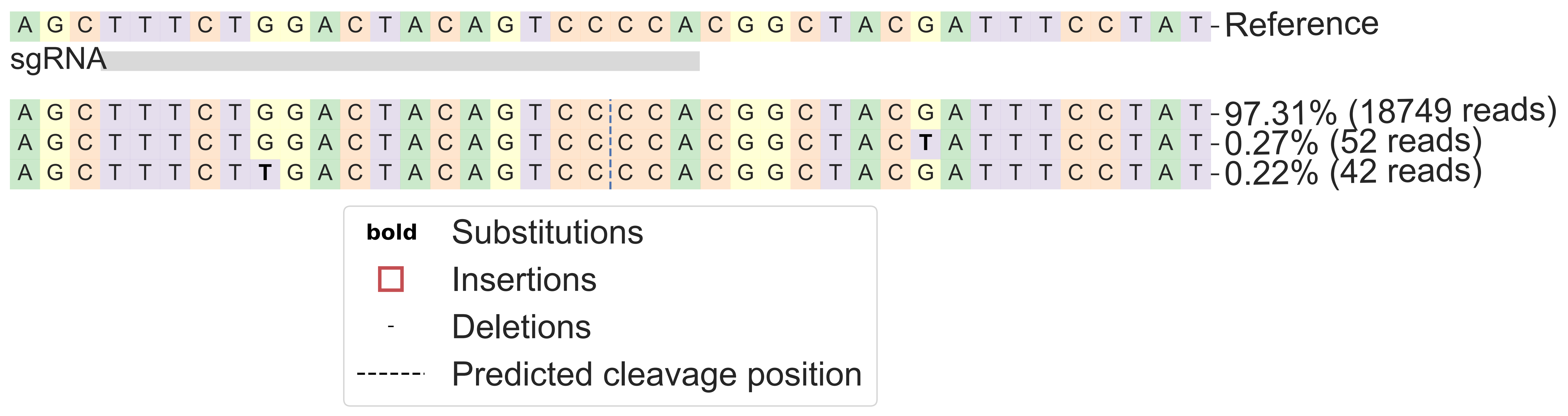
AGCTTTCTGGACTACAGTCCCCACGGCTACGATTTCCTAT Reference
sgRNA
AGCTTTCTGGACTACAGTCCCCACGGCTACGATTTCCTAT 97.31% (18749 reads)
AGCTTTCTGGACTACAGTCCCCACGGCTACTATTTCCTAT 0.27% (52 reads)
AGCTTTCTTGACTACAGTCCCCACGGCTACGATTTCCTAT 0.22% (42 reads)
bold Substitutions
Insertions
Deletions
Predicted cleavage position
